# Increased *whi*B7 expression and antibiotic resistance in *Mycobacterium chelonae* carrying two prophages

**DOI:** 10.1186/s12866-021-02224-z

**Published:** 2021-06-09

**Authors:** Jaycee Cushman, Emma Freeman, Sarah McCallister, Anna Schumann, Keith W. Hutchison, Sally D. Molloy

**Affiliations:** 1 Department of Molecular and Biomedical Sciences, University of Maine, Orono, ME United States; 2 The Honors College, University of Maine, Orono, ME United States

**Keywords:** Prophage, Antibiotic resistance, Mycobacteria, *whi*B7, Polymorphic toxin

## Abstract

**Background:**

The global rise in the incidence of non-tuberculosis mycobacterial infections is of increasing concern due their high levels of intrinsic antibiotic resistance. Although integrated viral genomes, called prophage, are linked to increased antibiotic resistance in some bacterial species, we know little of their role in mycobacterial drug resistance.

**Results:**

We present here for the first time, evidence of increased antibiotic resistance and expression of intrinsic antibiotic resistance genes in a strain of *Mycobacterium chelonae* carrying prophage*.* Strains carrying the prophage McProf demonstrated increased resistance to amikacin. Resistance in these strains was further enhanced by exposure to sub-inhibitory concentrations of the antibiotic, acivicin, or by the presence of a second prophage, BPs. Increased expression of the virulence gene, *whi*B7, was observed in strains carrying both prophages, BPs and McProf, relative to strains carrying a single prophage or no prophages.

**Conclusions:**

This study provides evidence that prophage alter expression of important mycobacterial intrinsic antibiotic resistance genes and additionally offers insight into the role prophage may play in mycobacterial adaptation to stress.

## Background

Prophage (integrated viral genomes) are major drivers of bacterial virulence and antibiotic resistance in bacteria, yet the mechanisms of prophage-mediated antibiotic resistance are unknown [1, 2]^.^ Prophages are common in mycobacteria, including clinical isolates of emerging non-tuberculosis pathogenic mycobacteria, *Mycobacterium avium* and *M. abscessus* [3, 4]. *M. abscessus* is of significant concern as it is considered one of the most antibiotic resistant pathogens [5, 6]. Extensively resistant isolates share increased expression of conserved mycobacterial intrinsic antibiotic resistance genes such as *whi*B7, making drug treatment challenging [5–7]*.* Understanding how intrinsic antibiotic resistance genes are regulated in pathogenic mycobacteria would provide opportunities to develop novel and more effective treatment approaches [7, 8].

Clarithromycin (CLA) combined with amikacin (AMK) is the treatment of choice for* M. abscessus* infections but with the emergence of resistance to both of these drugs, treatment is becoming increasingly difficult [7, 9]. Recent efforts in sequencing of clinical *M. abscessus* isolates determined that mutations in the 23S rRNA gene, *rrl,* and in the *erm* gene, which confers macrolide (e.g. clarithromycin) resistance, are typically associated with elevated CLA resistant phenotypes but did not account for all the clarithromycin resistant phenotypes [7]. What was consistent in all clarithromycin-resistant isolates was elevated expression of genes in the *whi*B7 regulon, including the transcription factor *whi*B7 and its target genes, the drug efflux pumps plus other antibiotic resistance genes including *erm* and *eis* [7, 10–12]. These latter genes confer macrolide and aminoglycoside resistance, respectively. Likewise, in AMK-resistant isolates of *M. abscessus* there is typically a 10-fold increase in *whi*B7 expression relative to AMK susceptible strains [13]. *whi*B7 is conserved across all mycobacteria, including pathogenic and non-pathogenic species such as *M. tuberculosis*, *M. abscessus*, *M. chelonae* and *M. smegmatis* [14, 15]. Characterizing the pathways that lead to increased *whi*B7 expression and intrinsic drug resistance in pathogenic mycobacteria will be important for identifying new targets for novel drug development [9, 16, 17].

The majority of bacterial pathogens carry prophage that are known to contribute to bacterial virulence and fitness [2, 18, 19]. Prophage introduce novel genes into bacterial genomes that can result in phenotypes that are more competitive in bacterial populations [2, 18]. Prophage also contribute to antibiotic resistance and persistence. Nine cryptic prophages (transcriptionally active prophage that cannot carry out lytic infections) in *E. coli* significantly increase resistance to quinolones and beta-lactam antibiotics compared to strains in which all or combinations of prophages had been cured, although the mechanism by which these prophage affected resistance was not reported [1]. Toxin/antitoxin (TA) systems encoded by prophage are also known to increase resistance and persistence in the presence of antibiotics. In *E. coli,* prophage-encoded TA pair RalR/RalA increases resistance to broad-spectrum fosfomycin and the RelE toxin of prophage Qin leads to persistence in the presence of ciprofloxacin, ampicillin and tobramycin [20–22]. The majority of mycobacterial pathogens also carry prophage and they are hypothesized to play a role in virulence, yet remain largely uninvestigated [3, 23, 24].

In this study we examine the impact of two mycobacteriophages on intrinsic antibiotic resistance and *whi*B7 expression in the non-tuberculosis mycobacterial pathogen, *M. chelonae,* a member of the *M. abscessus/chelonae* complex. The disease caused by *M. chelonae*, mainly soft tissue and disseminating infections, differs from that of *M. abscessus*; however, the *whi*B7-dependent mechanisms of intrinsic resistance are conserved across all mycobacteria, including *M. chelonae* [15, 25]. We identified a naturally occurring prophage in *M. chelonae* that also occurs in the sequenced genomes of at least 25 clinical isolates of *M. abscessus*. We characterized the genome of the *M. chelonae* prophage, McProf, and created a cured strain that lacks prophage. Antibiotic resistance and gene expression of this strain was compared to that of *M. chelonae* carrying a single or multiple prophages.

## Results

### Double lysogens of *M. chelonae* have increased resistance to aminoglycosides amikacin and tobramycin

The wild type *M. chelonae* (ATCC 35752) carries a naturally occurring 67,657-bp prophage that we have named McProf. To determine how prophages impact gene expression and the antibiotic resistance phenotype of *M. chelonae* we added a second prophage. We identified three mycobacteriophages capable of infecting *M. chelonae*, Muddy, WildCat and BPs, of which only BPs is known to be temperate [26–28]. A double lysogen of *M. chelonae* was created from the WT *M. chelonae* strain using the cluster G mycobacteriophage, BPs [28]. BPs integrates into an *attB* site located within the 3′ end of the host tRNA-Arg gene (BB28_RS01100), that is similar to the BPs *attB* site in *M. smegmatis* (Msmeg_6349) [28]. BPs lysogens of the *M. chelonae* WT strain (BPs, McProf) appear to be more stable than BPs lysogens of *M. smegmatis*. Lysogens form at a higher efficiency in *M. chelonae* WT (25%) compared to that in *M. smegmatis* (5%) and release fewer particles into cell culture supernatant (10^4^–10^5^ PFUs ml^− 1^ compared to 10^10^ PFUs ml^− 1^) [28, 29].

To determine if the presence of a second prophage in *M. chelonae* alters susceptibility to antibiotics, we determined the minimum inhibitory concentrations (MIC) for the double *M. chelonae* lysogen (BPs, McProf) relative to the WT strain (McProf) in the presence of varying levels of the aminoglycosides, amikacin (AMK) and tobramycin (TOB), and tetracycline (TET) (Table 1). The presence of the second prophage, BPs, significantly increased resistance to both aminoglycosides. There was not a consistent significant difference in resistance to TET. As a positive control, we exposed the *M. chelonae* (McProf) strain to sub-inhibitory concentrations of acivicin (ACI), a known inducer of intrinsic resistance in mycobacteria [12]. As expected, ACI significantly increased the resistance of *M. chelonae* (McProf) in both the AMK and TOB assays (Table 1). ACI treatment did not significantly alter TET resistance of *M. chelonae* (McProf).

**Table 1:** Minimum inhibitory concentrations of *M. chelonae* strains carrying one, two or no prophage in the presence and absence of ACI treatment

Strain (prophage):	MIC^a^ (μg ml^-1^)							
Antibiotic:	AMK		TOB		TET		CLA	
ACI treatment:	-ACI	+ACI	-ACI	+ACI	-ACI	+ACI	-ACI	+ACI
WT (McProf)	64	128	8	16	16	16	3	6
WT (McProf, BPs)	128	128	16	ND	16	16	3	ND
∆McProf	32	64	8	8	16	16	3	6
∆McProf (BPs)	32	64	8	ND	16	16	3	ND

^a^MIC determined as the lowest drug concentration that completely inhibited growth

**Fig. 1:**
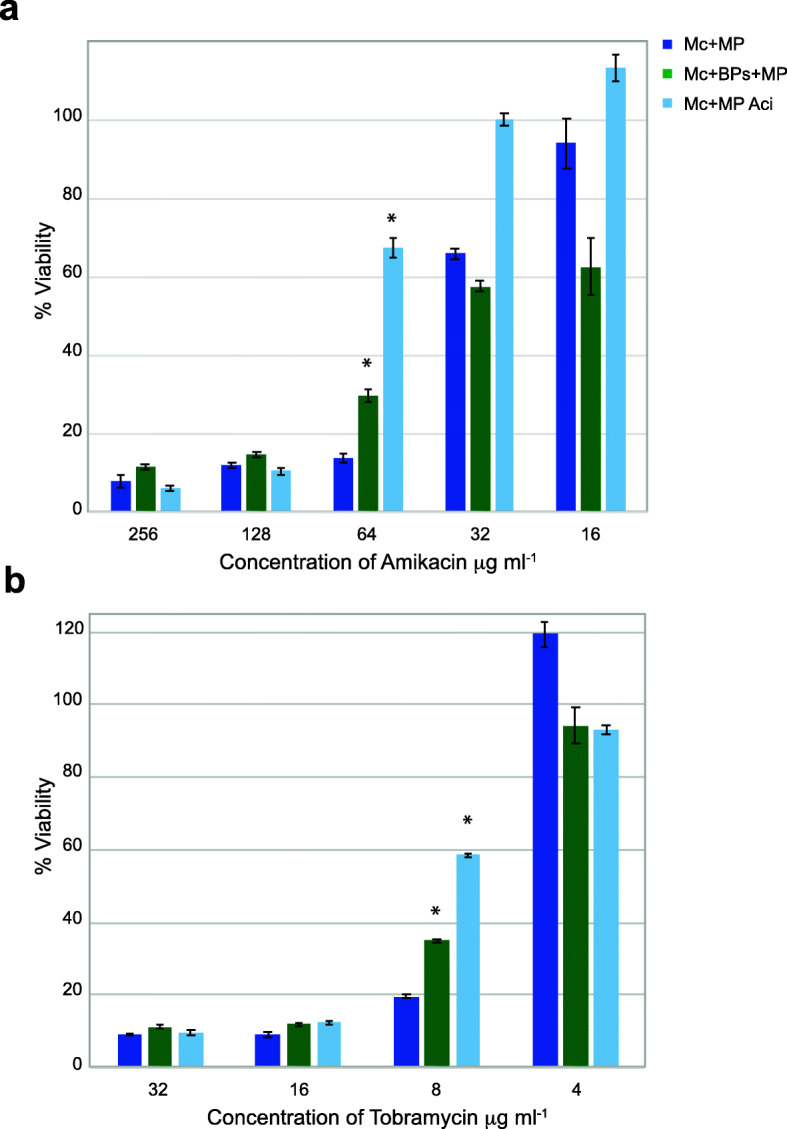
Percent viability of *M. chelonae* carrying single prophage McProf (MP) and two prophages BPs and McProf in the presence of varying concentrations of a. AMK and b. TOB. As a positive control, single McProf lysogens were treated with 75 μM acivicin (Aci), a known inducer of *whi*B7. Graphs represent average values ± SE of the mean with *n* = 6. The optical density was measured at 570- and 600 nm after addition of 1 μl of AlamarBlue and the percent difference in reduction between antibiotic-treated cells and untreated cells was calculated. Mean percent reduction by *M. chelonae* that are statistically significant are indicated by an Asterix (Wilcoxon rank sum, *p* < 0.05). Data is representative of three independent experiments

We also determined the viability of the strains after antibiotic treatment by adding AlamarBlue to the wells to detect metabolic activity (Fig. 1). There was a statistically significant difference in viability between the double lysogen and WT strains treated with 64 μg ml^− 1^ AMK and 8 μg ml^− 1^ TOB. We noted a slight increase in viability of the WT strain at 8 μg ml^− 1^ TOB; however, there was no evidence of growth at this concentration of TOB. The WT strain treated with ACI in both the AMK and TOB assay had the highest viability at these drug concentrations. Some background reduction of AlamarBlue was observed for both strains at doses higher than the observed MIC; however, there was no growth detected in those wells.

### Isolation of a non-lysogen and single BPs lysogen of *M. chelonae*

To better understand how the presence of the second prophage increases antibiotic resistance, we generated a strain of *M. chelonae* that contains no prophage (*M. chelonae* (ΔMcProf)) and from that a single BPs lysogen of *M. chelonae*. To remove the McProf prophage we created a recombinant strain of *M. chelonae* (McProf) that overexpresses the McProf excise gene, gp5, from an inducible mycobacterial expression plasmid (Table 2) [30]. Using sets of PCR primers that amplify either the bacterial attachment site (*attB*) and the phage attachment site (*attP*) or the hybrid prophage attachment sites, *attL* and *attR*, we identified ATc-induced bacterial colonies that had an intact *attB* site, indicating that the McProf prophage had been lost and that McProf has an active integrase system (Table 3) (data not shown). To determine if McProf phage particles are released from *M. chelonae* (McProf) cells through spontaneous induction, concentrated culture supernatants were plated onto lawns of the newly acquired non-lysogen strain (ΔMcProf), but we were unable to detect plaques. PCR analysis of *M. chelonae* (McProf) culture supernatants also failed to detect the McProf *attP* sequence, which would have indicated the presence of either excised McProf genome or linear McProf genome in phage particles. It is possible that there is a mutation that we were not able to identify that prevents McProf from carrying out a successful lytic infection. Alternatively, *M. chelonae* may not be the natural host and McProf is capable of lytically infecting other mycobacterial hosts.

**Table 2:** Bacterial strains and plasmids used in this study

Strains	Strain Description	Source
*Escherichia coli*		
DH5⍺	Supercompetent cells	NEB
*M. chelonae* WT	Laboratory strain of *M. chelonae* ATCC 35752 with naturally-occurring prophage, McProf	
*M. chelonae* double lysogen	M. chelonae +McProf +BPs	This study
*M. chelonae* nonlysogen	*M. chelonae* ΔMcProf	This study
*M. chelonae* BPs single lysogen	M. chelonae ΔMcProf+BPs	This study

**Table 3:** PCR primers used in this study

Description	Primers	Sequence (5' to 3')	Tm (˚C)	% GC	amplicon size (bp)
Primers used to detect McProf phage attachment sites in *M. chelonae*	Mc_attL_F	CGTCACGTTGGGGACTATCT	56.5	55	212
	Mc_attL_R	TTGAGCTGCGGATAACCTCT	56	50	
	Mc_attR_F	CGCTTGTAATCGTCGTCGTA	54.7	50	1067
	McProphageRR	ATAACTTTCGGCGGTTCCTT	54.5	45	
Primers used to detect BPs phage attachment sites in *M. chelonae*	BPs_attP_L	GCTTTATCCAGGGTTGACCA	54.8	50	203
	BPs_attP_R	GTTCCGATTAGTTGGCTGGA	54.8	50	
	BPs_attB_L	GTCTCGTTACTGGCGAGCTT	57.1	55	548
	BPs_attB_R	CGGGTAGTAGGCAGATGAGC	57.2	60	

The non-lysogen strain of *M. chelonae* (ΔMcProf) was used to isolate single lysogens of BPs. Although we were able to isolate BPs lysogens in the non-lysogen strain of *M. chelonae*, they are less stable than BPs lysogens formed in the WT strain (McProf) and comparable to lysogens formed in *M. smegmatis* [28, 29]. Lysogens formed at an efficiency of ~ 5%, and the titer of BPs in lysogen culture supernatants was 10^10^ PFUs ml^− 1^, several orders of magnitude higher than that of the double lysogen (10^5^ PFUs ml^− 1^).

### Single and double lysogens carrying McProf have higher AMK resistance than strains that lack McProf

**Fig. 2:**
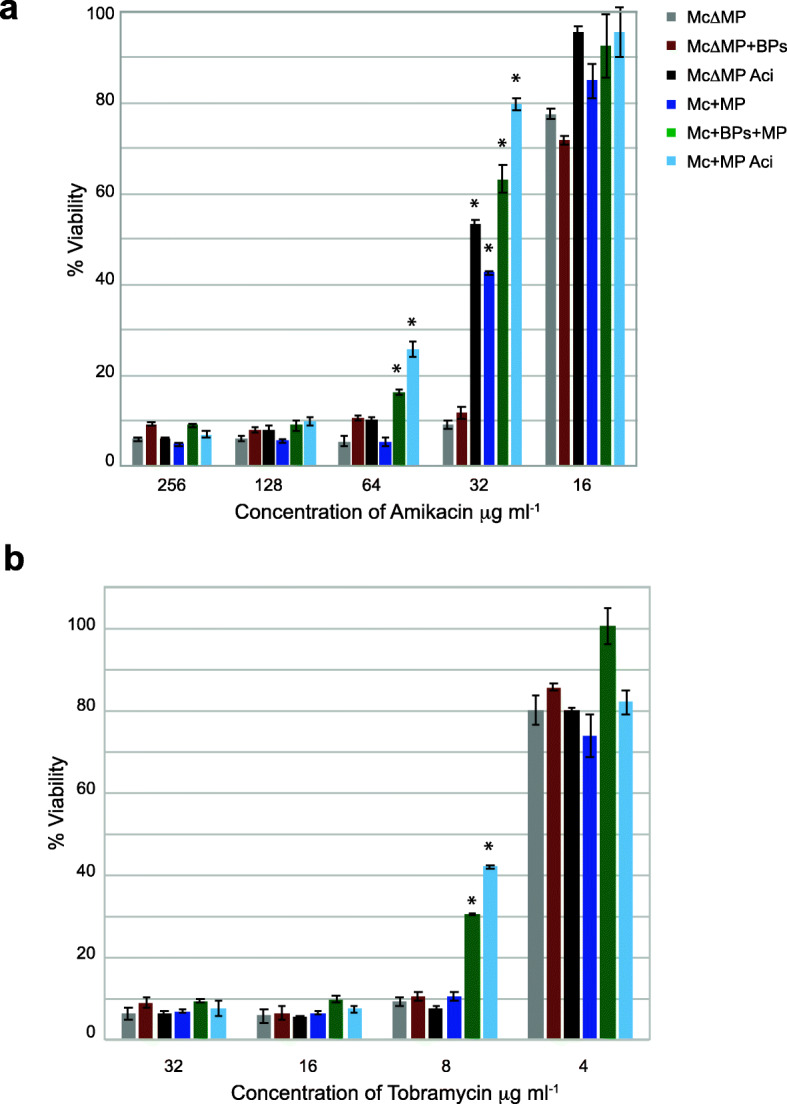
Percent viability of *M. chelonae* carrying single prophage McProf, two prophages BPs and McProf, no prophage, or single BPs prophage in the presence of varying concentrations of **a.** AMK and **b**. TOB. As a positive control, single McProf lysogens and non-lysogen cultures were treated with 75 μM acivicin (Aci), a known inducer of *whi*B7. Graphs represent average values ± SE of the mean with n = 6. The optical density of was measured at 570- and 600 nm after addition of 1 μl of AlamarBlue and the percent difference in reduction between antibiotic-treated cells and untreated cells was calculated. Mean percent reduction by *M. chelonae* that are statistically significant are indicated by an Asterix (Wilcoxon rank sum, p < 0.05). Data is representative of three independent experiments

To determine the roles of prophages BPs and McProf in the increased resistance observed in the double lysogen, we determined the MIC and viability of double (BPs, McProf) and single (BPs or McProf) *M. chelonae* lysogens relative to non-lysogen cells (ΔMcProf) in the presence of varying levels of AMK, TOB, TET and CLA (Table 1, Fig. 2). The presence of the naturally occurring prophage, McProf, significantly contributes to AMK resistance in *M. chelonae* in the presence and absence of ACI treatment (Table 1). The WT strain carrying McProf alone had a higher MIC for AMK (64 μg mL^− 1^) relative to the non-lysogen strain (ΔMcProf) (MIC of 32 μg mL^-1)^ [10, 12]. Treatment of these two strains with ACI increased the MIC for both strains; however, the MIC for ACI-treated WT (McProf) strain was two-fold higher than that of the ACI-treated non-lysogen (ΔMcProf) strain (Table 1.) The presence of a second prophage, BPs, also increases resistance to AMK, with bacterial growth at doses as high as 64 μg mL^− 1^. Although the double lysogen had the same MIC as the ACI-treated WT strain (128 μg mL^− 1^) (Table 1), the cell viability of the ACI-treated WT strain was statistically higher than that of the double lysogen (Fig. 2a). BPs alone had no effect on AMK resistance suggesting that BPs only increases AMK resistance through an interaction with the naturally occurring prophage, McProf.

The presence of the prophages McProf and BPs also altered TOB resistance in *M. chelonae* (Table 1 and Fig. 2b). The WT (McProf) strain did not have a higher TOB MIC than the non-lysogen strain (8 μg mL^− 1^), but in the presence of ACI, the MIC for WT (McProf) strain increased two-fold (16 μg mL^− 1^), whereas the MIC for the non-lysogen (ΔMcProf) strain remained 8 μg mL^− 1^. It is possible a difference would be detected at TOB doses between 4- and 8 μg mL^− 1^. The TOB MIC for the double lysogen (BPs, McProf) was the same as that for the ACI-treated WT (McProf) strain; however the viability for ACI-treated WT (McProf) strain was significantly higher than that of the double lysogen at 8 μg mL^− 1^ (Table 1 and Fig. 2b).

The presence of prophages had little effect on TET and CLA resistance in *M. chelonae* (Table 1). ACI treatment induced significant increases in CLA resistance but not TET resistance in the WT (McProf) and non-lysogen (ΔMcProf) strains.

### Prophage McProf enhances AMK resistance in response to sub-inhibitory concentrations of antibiotics

**Fig. 3:**
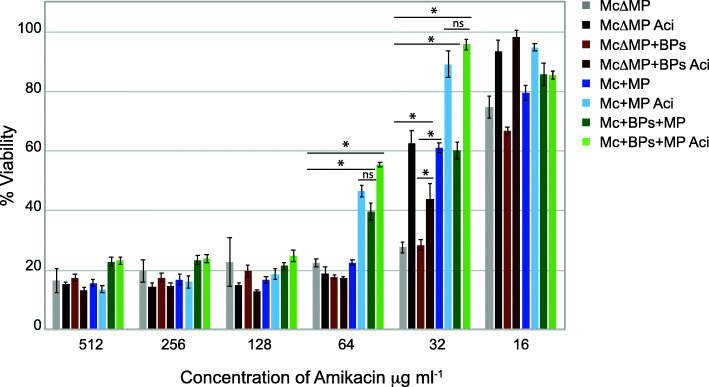
Percent viability of *M. chelonae* carrying no prophage ΔMcProf (McΔMP), BPs prophage (Mc + BPsΔMP), prophage McProf (Mc + MP) or both prophages BPs and McProf (Mc + BPs + MP) in the presence of varying concentrations of amikacin. To determine if the presence of each prophage interacts with sub-inhibitory concentrations of antibiotics, each strain was treated or not treated with 75 μM acivicin (ACI). Graphs represent average values ± SE of the mean with *n* = 3. The optical density of was measured at 570- and 600 nm after the addition of 2 μl of AlamarBlue and the percent difference in reduction between antibiotic-treated cells and untreated cells was calculated. Data is representative of two independent experiments

Because the *M. chelonae* (McProf) strain treated with ACI had higher AMK resistance than the non-lysogen strain treated with ACI, we wondered if the presence of prophage McProf enhances the effect of sub-inhibitory concentrations of antibiotics on AMK resistance. To determine the interaction between ACI and the presence of one or both prophages, we treated all four lysogen and non-lysogen strains with sub-inhibitory concentrations of ACI and repeated the AMK resistance assay. The presence of McProf increases the effect of ACI on AMK resistance compared to the non-lysogen whereas the BPs prophage alone does not (Table 1 and Fig. 3). The double lysogen treated with ACI did not have a higher MIC than the WT (McProf) strain treated with ACI; however, cultures of the ACI-treated double lysogen (BPs, McProf) did consistently have a statistically higher viability than that of ACI-treated WT (McProf) strain at AMK doses of 64 μg mL^− 1^. The presence of BPs and ACI appear to interact with McProf to increase AMK resistance.

### The *whi*B7 regulon is upregulated in double lysogens of *M. chelonae*

**Fig. 4:**
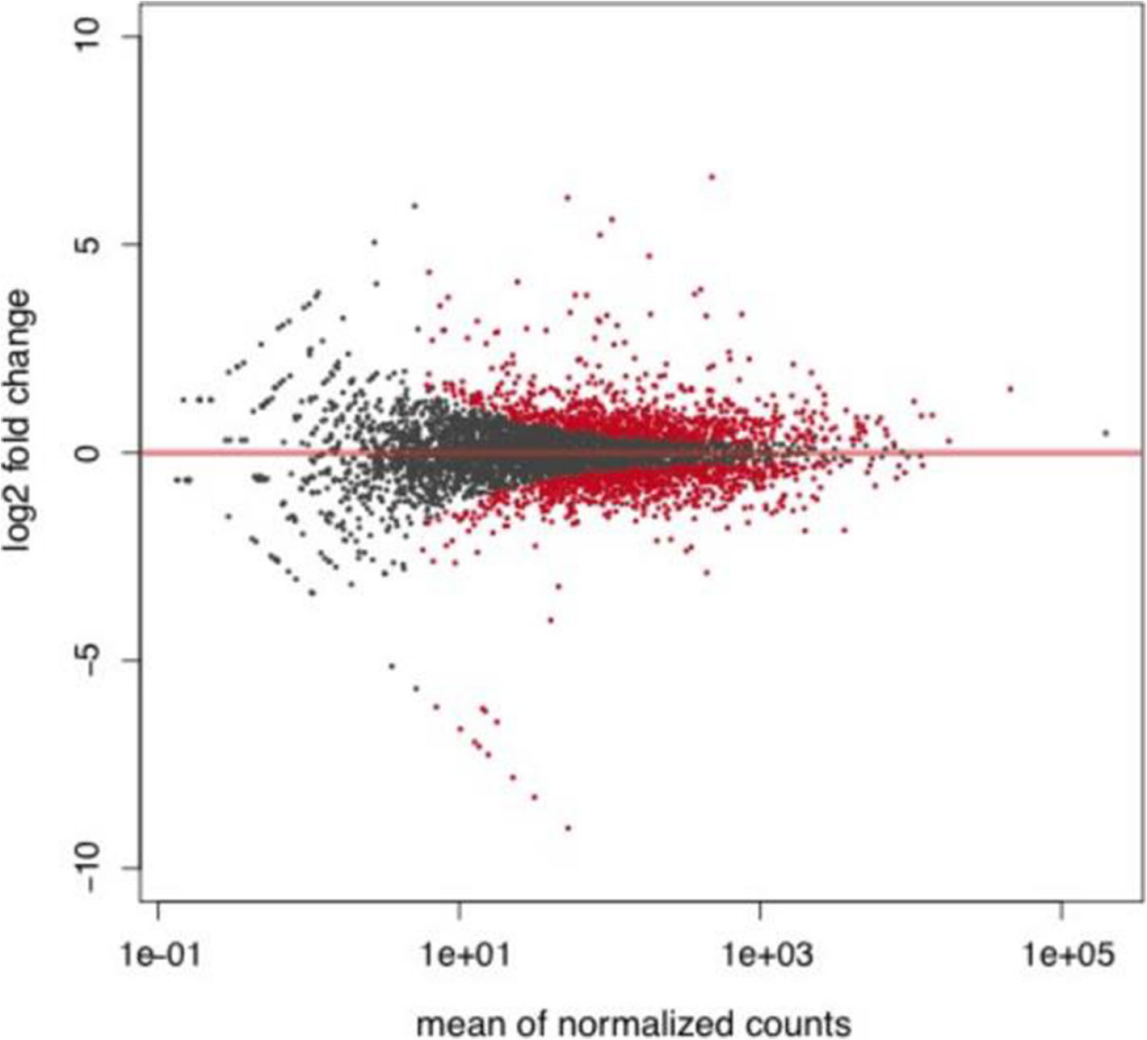
MA Plot presenting the relationship between average expression level (mean of normalized counts) of *M. chelonae* genes and their fold change (log2fold change) in the double *M. chelonae* lysogen (BPs, McProf) relative to the WT strain (McProf). Red indicates genes identified as differentially expressed at an FDR of 0.05 or smaller

RNAseq analysis was performed on RNA isolated from the WT (McProf) and double lysogen (BPs, McProf) *M. chelonae* strains to learn if the presence of the second prophage, BPs, impacted expression of genes that may be involved in mycobacterial antibiotic resistance. The presence of prophage BPs significantly altered expression of *M. chelonae* genes, including numerous putative virulence genes. Out of 4867 genes in the *M. chelonae* genome, 417 (8.5%) were differentially regulated in the double lysogen.

(BPs, McProf) (Fig. 4).

The majority of the top-ranked genes in the double lysogen belonged to the *whi*B7 regulon, genes in *M. tuberculosis* with functions related to antibiotic resistance and increased survival in macrophage (Tables 4 and 5) [15]. The transcription factor, identified as *whi*B7 (BB28_RS17590)*,* was the fifth most highly upregulated gene in the double lysogen with a fold change of 26.5 (Log2FC = 4.7, FDR = 1.3^− 73^) (Table 4). The WhiB7 peptide sequence shares 95% identity with the *M. abscessus* WhiB7 peptide (MAB_3508c) and has all the conserved residues that form the iron sulfur cluster binding domain. It also has the glycine-rich motif of the signature WhiB7 C-terminal “A/T Hook” DNA binding domain, which binds to AT-rich sequences adjacent to target gene promoters [11, 12]. The *M. chelonae* genome contains a large *whi*B7 regulon like that of *M. abscessus*, with 103 of the 128 *whi*B7 regulon genes found in *M. abscessus* [12]. We identified a total of 30 upregulated genes that belong to the *whi*B7 regulon, many of which are known to contribute to drug resistance such as GNAT acetyltransferases, *eis*1 (BB28_RS05390) and *eis*2 (BB28_RS22650), multi-drug efflux transporter *tap* (BB28_RS06750), and the *tet*V efflux pump (BB28_RS13560). Also included in this regulon are additional GNAT acetyltransferases (BB28_RS23100 and BB28_RS01940) and ABC transporters with ATP binding domains that likely function in drug resistance (Tables 4 and 5). In *M. abscessus* and *M. tuberculosis, erm* is part of the *whi*B7 regulon and provides macrolide resistance but the gene is not present in the *M. chelonae* genome [31]. *M. chelonae* lacks this *whi*B7 regulon gene, but it does encode a newly discovered gene in the *whi*B7 regulon*,* the ribosome splitting factor *hflX* (MAB_3042c)*,* which is reported to contribute to macrolide resistance in *M. abscessus* [31, 32]. Expression of the *M. chelonae hflX* was slightly elevated in double lysogens relative to the WT strain (McProf) (BB28_RS14985; Log2FC = 1.5, FDR = 8.4^− 33^) but this did not result in significant changes in CLA resistance in the double lysogen (Table 5). An additional 25 *whi*B7 regulon genes were upregulated but had fold changes of less than 2.

**Table 4:** Top 20 upregulated and downregulated *M. chelonae* genes in the double lysogen (BPs, McProf) relative to WT *M. chelonae* (McProf)

MCH^a^ gene ID	Predicted function^b^	log2FC^c^	FDR^d^	MAB^e^ Gene ID	MAB Gene Description	MTB^f^ Gene ID	MTB Gene Description
BB28_RS04255	flotillin protein	6.6	6.4E-124	NA	NA	NA	NA
BB28_RS19005	hypothetical protein	6.1	9.1E-18	MAB_3786c	Hypothetical protein	NA	NA
**BB28_RS23100**	**N-acetyltransferase**	5.6	4.4E-38	MAB_4621c	Putative acetyltransferase	NA	NA
**BB28_RS23095**	**hypothetical protein**	5.3	2.8E-35	MAB_4620c	Hypothetical protein	NA	NA
**BB28_RS17590**	**WhiB7 transcriptional regulator**	4.7	1.3E-73	MAB_3508c	Putative transcriptional regulator	Rv3197A	transcriptional regulator WhiB7
**BB28_RS05390**	**GNAT family N-acetyltransferase (eis1)**	4.1	5.8E-12	MAB_1125c	Hypothetical acetyltransferase, GNAT family	Rv1947	hypothetical protein
BB28_RS04260	class I SAM-dependent methyltransferase	3.9	1.0E-120	MAB_0963c	Putative polyketide synthase protein	NA	NA
BB28_RS03915	hypothetical protein	3.8	2.8E-116	MAB_0857	Putative monooxygenase	NA	NA
BB28_RS20465	KR domain-containing protein	3.8	3.4E-26	MAB_4053c	Putative short chain dehydrogenase/reductase	NA	NA
BB28_RS16665	iron ABC transporter permease	3.8	4.5E-31	MAB_2262c	Hypothetical ABC transporter ATP-binding protein	Rv1348	iron ABC transporter ATP-binding protein/permease IrtA
**BB28_RS06460**	**NAD(P)-dependent oxidoreductase**	3.4	1.2E-21	MAB_1344c	Putative DTDP-glucose-4,6-dehydratase-related protein	Rv3468c	dTDP-glucose 4,6-dehydratase
**BB28_RS11540**	**ABC transporter ATP-binding protein**	3.3	3.5E-64	MAB_2355c	Putative ABC transporter ATP-binding protein	NA	NA
**BB28_RS22650**	**GNAT family N-acetyltransferase (eis2)**	3.3	1.4E-176	MAB_4532c	hypothetical protein	NA	NA
**BB28_RS06750**	**tap multidrug efflux transporter**	3.3	7.3E-35	MAB_1409c	Putative drug antiporter protein precursor	Rv1258c	multidrug-efflux transporter
**BB28_RS13560**	**TetV Efflux Pump**	3.3	9.8E-94	MAB_2780c	Putative transporter	NA	NA
**BB28_RS20470**	**pyridoxamine 5'-phosphate oxidase family protein**	3.2	4.1E-29	MAB_4054c	hypothetical protein	NA	NA
BB28_RS03190	EamA family transporter	3.2	3.5E-06	MAB_0677c	Conserved hypothetical protein	NA	NA
**BB28_RS17050**	**hypothetical protein**	3.2	5.1E-32	MAB_3424c	hypothetical protein	NA	NA
BB28_RS06235	acyltransferase	3.1	2.7E-39	MAB_1297c	hypothetical protein	NA	NA
**BB28_RS01940**	**N-acetyltransferase**	3.0	2.9E-11	MAB_0404c	Putative acetyltransferase	NA	NA
BB28_RS13285	ferrochelatase	-2.1	4.5E-40	MAB_2721c	Ferrochelatase (Protoheme ferro-lyase)	Rv1485	ferrochelatase
BB28_RS19235	alpha-hydroxy-acid oxidizing enzyme	-2.1	2.9E-35	MAB_3834c	Possible L-lactate dehydrogenase (cytochrome) LldD1	Rv0694	mycofactocin system heme/flavin oxidoreductase MftD
BB28_RS08645	epoxide hydrolase	-2.2	2.4E-07	MAB_1628c	hypothetical protein	NA	NA
BB28_RS13295	beta-ketoacyl-ACP reductase	-2.3	3.4E-56	MAB_2723c	3-oxoacyl-	Rv1483	3-oxoacyl-ACP reductase FabG
BB28_RS13290	enoyl-acyl-carrier-protein reductase FabI	-2.4	2.0E-60	MAB_2722c	Enoyl-(acyl-carrier-protein) reductase (NADH)	Rv1484	NADH-dependent enoyl-ACP reductase
BB28_RS08640	hypothetical protein	-2.4	2.6E-04	NA	NA	NA	NA
BB28_RS19655	universal stress protein	-2.7	1.2E-03	MAB_3904	hypothetical protein	Rv2028c	universal stress protein
BB28_RS16545	acyl-ACP desaturase	-2.9	1.3E-75	MAB_3354	Probable acyl-	NA	NA
BB28_RS05070	cytochrome c oxidase subunit I	-3.2	2.8E-16	MAB_1042c	Probable cytochrome c oxidase polypeptide I	NA	NA
BB28_RS11065	iron ABC transporter permease	-4.0	3.8E-16	NA	NA	NA	NA
BB28_RS01880	DUF58 domain-containing protein	-6.2	5.8E-06	MAB_0388c	hypothetical protein	Rv3693	membrane protein
BB28_RS01835	glycerol uptake glpF	-6.2	4.5E-06	MAB_0381	Glycerol uptake facilitator protein (GlpF)	NA	NA
BB28_RS01885	MoxR family ATPase	-6.5	1.2E-06	MAB_0389c	Putative regulatory protein	Rv3692	methanol dehydrogenase transcriptional regulator MoxR
BB28_RS01875	stage II sporulation protein M	-6.6	1.2E-06	MAB_0387	hypothetical protein	Rv3694c	transmembrane protein
BB28_RS24340	RDD family protein	-7.0	2.4E-07	MAB_0386c	hypothetical protein	Rv3695	membrane protein
BB28_RS01830	KR domain-containing protein	-7.1	1.5E-07	MAB_0380	Probable short-chain dehydrogenase/reductase	NA	NA
BB28_RS01890	DUF4350 domain-containing protein	-7.3	4.6E-08	MAB_0390c	hypothetical protein	Rv3691	hypothetical protein
BB28_RS01900	membrane protein	-7.8	2.0E-09	MAB_0392c	hypothetical protein	Rv3689	transmembrane protein
BB28_RS01845	PadR family transcriptional regulator	-8.3	1.4E-10	MAB_0383c	Putative transcriptional regulator, PadR-like	NA	NA
BB28_RS01840	glycerol kinase	-9.0	1.1E-12	MAB_0382	Glycerol kinase (GlpK)	Rv3696c	glycerol kinase

^a^MCH = *Mycobacterium chlelonae*

^b^Genes that belong to the WhiB7 regulon are in bold

^c^Log2 Fold Change for the double lysogen (+BPs+McProf) of *M. chelonae*

^d^FDR = False Discovery Rate

^e^MAB = *Mycobacterium abscessus*

^f^MTB = *Mycobacterium tuberculosis*

**Table 5:** WhiB7 regulon genes upregulated in *M. chelonae* double lysogens relative to the WT *M. chelonae* (McProf)

MCH^a^ gene ID	Predicted function	log2 FC^b^	FDR^c^	MAB^d^ gene ID	Predicted Function
BB28_RS19005	hypothetical protein	6.1	9.1E-18	MAB_3786c	Hypothetical protein
BB28_RS23100	N-acetyltransferase	5.6	4.4E-38	MAB_4621c	Putative acetyltransferase
BB28_RS23095	hypothetical protein	5.2	2.8E-35	MAB_4620c	Hypothetical protein
BB28_RS17590	WhiB7 transcriptional regulator	4.7	1.3E-73	MAB_3508c	Putative transcriptional regulator
BB28_RS05390	GNAT family N-acetyltransferase (eis1)	4.1	5.8E-12	MAB_1125c	Hypothetical acetyltransferase, GNAT family
BB28_RS06460	NAD(P)-dependent oxidoreductase	3.4	1.2E-21	MAB_1344c	Putative DTDP-glucose-4,6-dehydratase-related protein
BB28_RS11540	ABC transporter ATP-binding protein	3.3	3.5E-64	MAB_2355c	Putative ABC transporter ATP-binding protein
BB28_RS22650	GNAT family N-acetyltransferase (eis2)	3.3	1E-176	MAB_4532c	N- acetyltransferase( eis2)
BB28_RS06750	*tap* multidrig efflux transporter	3.3	7.3E-35	MAB_1409c	Putative drug antiporter protein precursor
BB28_RS13560	TetV Efflux Pump	3.3	9.8E-94	MAB_2780c	TetV Efflux Pump
BB28_RS20470	pyridoxamine 5'-phosphate oxidase family protein	3.2	4.0E-29	MAB_4054c	Hypothetical protein
BB28_RS17050	hypothetical protein	3.2	5.1E-32	MAB_3424c	hypothetical protein
BB28_RS01940	N-acetyltransferase	3.0	2.9E-11	MAB_0404c	Putative acetyltransferase
BB28_RS09285	ABC transporter ATP-binding protein	2.9	2.7E-07	MAB_1846	Putative ABC transporter ATP-binding protein
BB28_RS12530	18 kDa antigen (HSP 16.7)	2.3	2.5E-31	MAB_3467c	18 kDa antigen (HSP 16.7)
BB28_RS03635	EamA/RhaT family transporter	2.0	1.4E-04	MAB_0766	Hypothetical conserved integral membrane protein
BB28_RS18890	membrane protein	1.8	1.9E-17	MAB_3762	hypothetical protein
BB28_RS17595	hypothetical protein	1.8	3.6E-41	MAB_3509c	Hypothetical protein
BB28_RS06440	TIGR00730 family Rossman fold protein	1.6	2.7E-08	MAB_1340	hypothetical protein
BB28_RS13900	methyltransferase domain-containing protein	1.6	1.6E-13	MAB_2845	Probable trans-aconitate methyltransferase
BB28_RS05350	hypothetical protein	1.6	2.2E-27	MAB_1117c	Hypothetical protein
BB28_RS14985	GTPase HflX	1.5	8.4E-33	MAB_3042c	Probable GTP-binding protein HflX
BB28_RS20665	isocitrate lyase (AceA)	1.5	3.9E-12	MAB_4095c	Isocitrate lyase (AceA)
BB28_RS06230	hypothetical protein	1.5	2.7E-03	MAB_1296	hypothetical protein
BB28_RS06840	hypothetical protein	1.5	1.1E-05	MAB_1413	hypothetical protein
BB28_RS00915	cation transporter	1.5	4.6E-29	MAB_0183c	Putative cation transporter
BB28_RS19745	LysE family translocator	1.5	3.7E-07	MAB_3913	Putative translocator
BB28_RS06445	TIGR00730 family Rossman fold protein	1.4	2.1E-06	MAB_1341	hypothetical protein
BB28_RS12685	aminoglycoside phosphotransferase	1.1	1.6E-02	MAB_4837c	Possible phosphotransferase
BB28_RS14560	GNAT family N-acetyltransferase	1.0	2.7E-17	MAB_2959	Putative acetyltransferase

^a^MCH = *Mycobacterium chlelonae*

^b^Log2 Fold Change for the double lysogen (+BPs+McProf) of *M. chelonae*

^c^FDR = False Discovery Rate

^d^MAB = *Mycobacterium abscessus*

The most highly regulated gene, with a 99-fold increase in expression, was annotated as a flotillin protein with no known function (BB28_RS01845) (log2FC = 6.6, FDR = 6.4^− 124^) (Table 4). Several of the most down regulated genes in the lysogen include a *pad*R-family transcription factor (BB28_RS01835, Log2FC = − 8.3, FDR = 1.4^− 10^) and genes involved in glycerol uptake (*glp*F, BB28_RS01835, Log2FC = − 6.2, FDR = 4.5^− 06^) and metabolism (*glp*K, BB28_RS01840, Log2FC = − 9.0, FDR = 1.1^− 12^) (Table 4) [33].

### Upregulation of *whi*B7 only occurs in double lysogens of *M. chelonae*

**Fig. 5:**
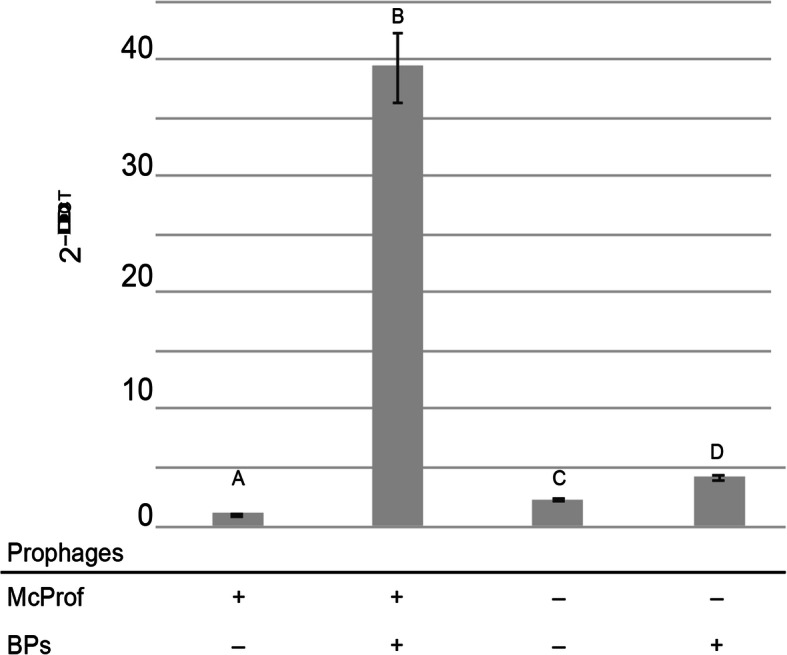
The average relative expression levels of *whi*B7 in *M. chelonae* carrying McProf alone, BPs and McProf, no prophage, and BPs alone as measured with SYBR Green quantitative RT-PCR. Cultures were grown to an OD_600_ of 1.0 before harvesting RNA in triplicate. Graphs represent average values ± standard error of the mean with n = 3 and are representative of two independent trials. Means with different letters are significantly different (Tukey’s HSD, α = 0.05)

To determine how the presence and absence of each prophage impacts *whi*B7 expression, *whi*B7 mRNA levels were measured by qPCR in the BPs single lysogen, double lysogen (BPs, McProf), and non-lysogen (ΔMcProf) and compared to that of the WT strain (McProf) (Fig. 5). Although *whi*B7 expression was slightly elevated in the non-lysogen (2-fold) and BPs single lysogen (4-fold) strains relative to the WT strain (McProf), the dramatic increase in *whi*B7 expression (~ 40-fold) only occurred in *M. chelonae* carrying both prophages (BPs, McProf). The elevated *whi*B7 expression occurred in the absence of known inducers of *whi*B7, such as ACI, which suggests BPs interacts with prophage McProf, resulting in *whi*B7 induction. The elevated expression of *whi*B7 in the double lysogen likely explains the increased resistance to AMK and TOB in the absence of ACI treatment (Table 1 and Fig. 2).

### Sub-lethal concentrations of ACI but not AMK induce *whi*B7 expression in the double lysogen of *M. chelonae*

**Fig. 6:**
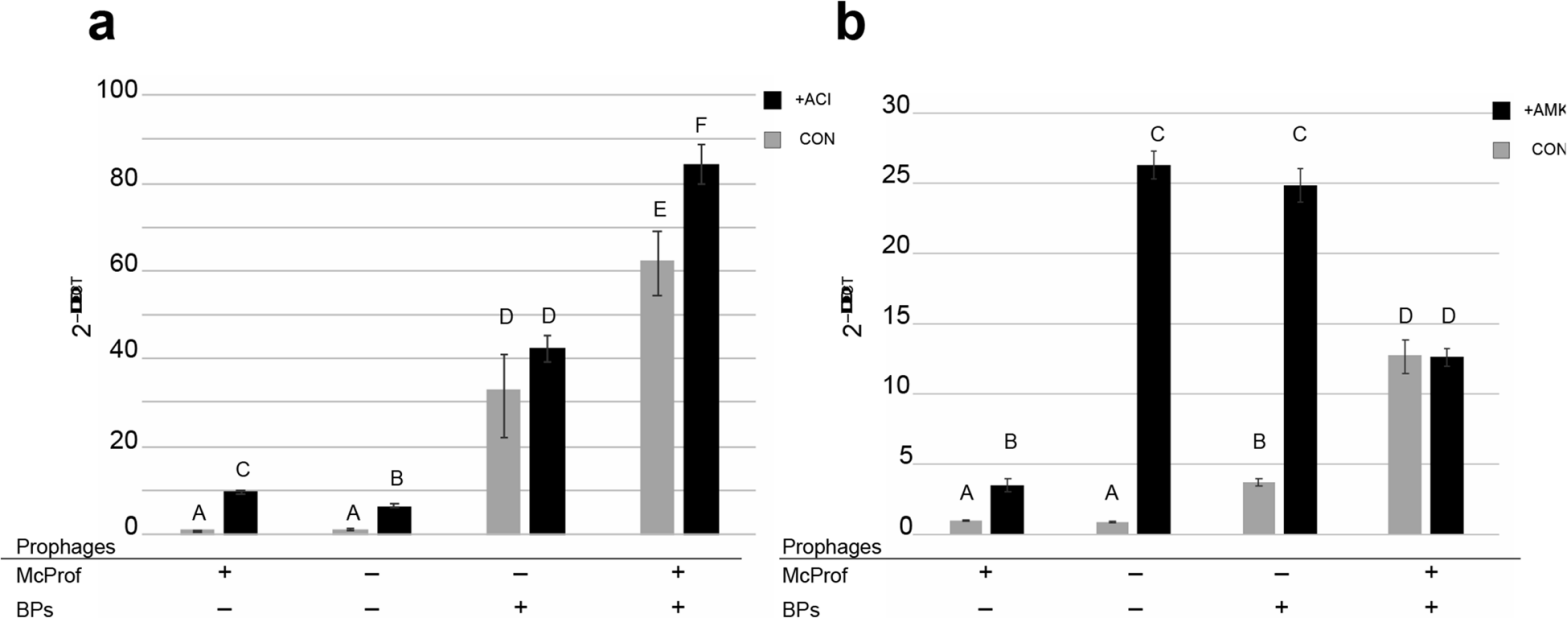
The average relative expression levels of *whi*B7 in *M. chelonae* carrying McProf alone, no prophage, BPs alone, or BPs and McProf as measured with SYBR Green quantitative RT-PCR. **a.** RNA was harvested in triplicate from strains grown to an OD_600_ of 0.7 before treating or not treating with 75 μM ACI and incubating for an additional 3 h. The culture OD_600_ at time of harvest was close to 1 but differed slightly for each strain and treatment. **b.** RNA was harvested in triplicate from strains grown to an OD_600_ of 0.9 before treating or not treating with 16.7 μM AMK and incubated for an additional 1 h. Graphs represent average values ± standard error of the mean with n = 3. Means with different letters are significantly different (Tukey’s HSD, α = 0.05)

We were surprised that the *M. chelonae* (McProf) strain had the lowest expression of *whi*B7 expression among the four strains given that it had higher AMK resistance, both in the presence and absence of ACI, than the two strains that lack McProf. We reasoned this may be due to *whi*B7-independent intrinsic resistance, such as cell wall permeability, and/or *whi*B7 induction in the presence of AMK, which is a more potent inducer of *whi*B7 than ACI [12]. Likewise, we wondered if the heightened viability observed in the single and double McProf lysogen strains in the presence of AMK and ACI was due to increased *whi*B7 expression. We therefore measured *whi*B7 expression in all four strains in the presence and absence of sublethal concentrations of ACI (75 μM) or AMK (16.7 μM) (Fig. 6).

ACI treatment resulted in increased *whi*B7 expression in all four strains relative to untreated strains (Fig. 6a). Expression of *whi*B7 was highest in the double lysogen strain (BPs, McProf) treated with ACI which correlates with the observed AMK resistance of this strain. *whi*B7 expression in the single and non-lysogen strain increased with ACI treatment; however, the relative levels of *whi*B7 expression did not correlate with AMK resistance (Fig. 2a). Although the fold-increase of *whi*B7 in ACI-treated strains relative to control strains was highest in the WT strain (McProf) (9.5-fold) *whi*B7 was lower than that of the ACI-treated BPs single lysogen, which demonstrated lower AMK resistance.

To determine if exposure to AMK also contributes to *whi*B7 expression in each of the four strains, *whi*B7 expression was determined in each of the strains in the presence and absence of sub-lethal concentrations of AMK (16.7 μM). Strains that lack the McProf prophage had the greatest increase in *whi*B7 expression in response to AMK treatment. The non-lysogen and BPs single lysogen had 28- and 7-fold increases in *whi*B7 expression in response to AMK treatment, respectively (Fig. 6b). AMK had less of an effect on *whi*B7 expression in strains carrying McProf. AMK treatment resulted in a 3.5-fold increase in *whi*B7 expression in the single McProf lysogen and no significant increase in *whi*B7 expression in the double lysogen. It’s possible that AMK doesn’t result in strong induction of *whi*B7 expression in the McProf-carrying strains due to cell wall permeability and/or efflux, and if so, this could also explain the AMK-resistant phenotypes observed in the single McProf and double lysogen strains.

### Organization of the McProf prophage genome

**Fig. 7:**
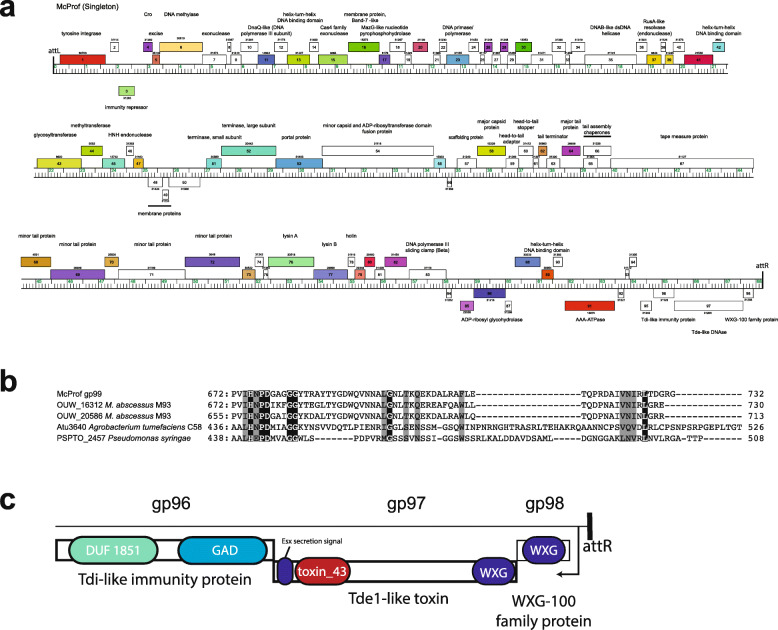
McProf genome map and TA system analysis. Genome organization of prophage McProf. a. The coordinates of the McProf genome are represented by the ruler. Genes are shown as colored boxes above (transcribed rightwards) or below (transcribed leftward) the ruler. The map was generated using Phamerator [34]. b. Partial alignment of McProf gp97 with representative members of the Tde superfamily that contain the toxin_43 domain and conserved HXXD catalytic motif. c. Graphical domain organization of McProf gp 96–98

To better understand how the two prophages, BPs, and McProf, may be interacting to alter *whi*B7 expression, we characterized the McProf genome and examined viral gene expression profiles from both McProf and BPs prophage genomes in the double lysogen. The McProf genome is 67,657 bp in length (*M. chelonae* CCUG 47445 coordinates 1,521,426 – 1,589,648) and encodes 98 putative genes and no tRNAs (Fig. 7a). The prophage genome is flanked by 45-bp phage attachment sites, *attL* and *attR* (5′- TGCGCCGTCAGGGGCTCGAACCCCGGACCCGCTGATTAAGAGTCA). The right attachment site, *attR*, overlaps a leftward oriented tRNA-Lys (BB28_RS07905). Located adjacent to the left attachment site, *attL*, is a rightward transcribed tyrosine integrase (gp1), one gene of unknown function (gp2) and a leftward transcribed gene, gp3, that is likely to be the immunity repressor, as it shares high amino acid sequence similarity with the immunity repressors of singleton mycobacteriophage DS6A (66%) and cluster K2 mycobacteriophages (70%) DismalFunk, DismalStressor, Findley, Marcoliusprime and Milly [35]. Gp4 and gp5 both have helix-turn-helix DNA binding motifs and encode Cro (control of repressor’s operator) and excise, respectively.

Located between *attR* and the structural genes (gp51–82) are genes that are typically expressed during lysogeny [36]. We were unable to predict a function for the majority of these genes; however, we were able to identify an ADP-riboysl glycosylhydrolase (gp86), a helix-turn-helix DNA binding protein (gp89), a membrane protein (gp90), and an AAA-ATPase (gp91). Most intriguing is the leftward transcribed gene cassette immediately adjacent to *attR*, which encodes proteins that may be secreted by the mycobacterial Type 7 secretion system (T7SS) (Esx-3 or Esx4) (Fig. 7b and c). Gp98 encodes a 105-amino acid gene product that forms four HHpred predicted helical domains with high probability matches to WXG-100 family motifs of T7SS proteins. The gp98 sequence contains a SAG motif, which strays slightly from the conserved WXG motif that is characteristic of T7SS secreted substrates [37]. Gp97 encodes a 732-residue polymorphic toxin that has a WXG-100 motif in the N-terminus and a possible T7SS secretion signal (YxxxD/E) in the C-terminus [38]. The C-terminus also includes a toxin_43 motif (PF15604.6) and high sequence similarity to the C-terminus of Type 6 secretion system (T6SS) polymorphic toxin, TdeI (Atu4350), found in *Agrobacterium tumefaciens* [39]. This family of proteins has DNAse activity and shares a conserved HXXD catalytic domain located in the C-terminus (Fig. 7b) [39]. Tde toxins are typically paired with a Tdi immunity protein and a likely immunity protein, gp96, was identified downstream of McProf gp97. McProf gp96 encodes a putative 216-residue protein that contains GAD-like and DUF1851 domains, which are well-conserved domains of Tdi homologs (Fig. 7c) [39].

Although the McProf prophage was identified and characterized in *M. chelonae*, it is closely related to prophages found in the genome sequences of clinical *M. abscessus* isolates. BlastN analysis of the McProf prophage genome in *M. abscessus*-specific databases (e.g. phagesdb.org) identified 25 *M. abscessus* isolates with McProf-like prophage sequences [35]. The WXG-100 family polymorphic toxin cassette identified in McProf is also prevalent in *M. abscessus* genomes. BlastP analysis of the McProf Tde-like polymorphic toxin (gp97 toxin) results in 100 high-similarity protein alignments to mycobacterial proteins with 91% matching *M. abscessus* sequences. An initial random screen of 10 of the aligned sequences showed that they were all located in prophage genomes flanked by a WXG-100 family gene and an immunity gene.

### Lysogenic gene expression profiles from the BPs and McProf prophage genomes

**Fig. 8:**
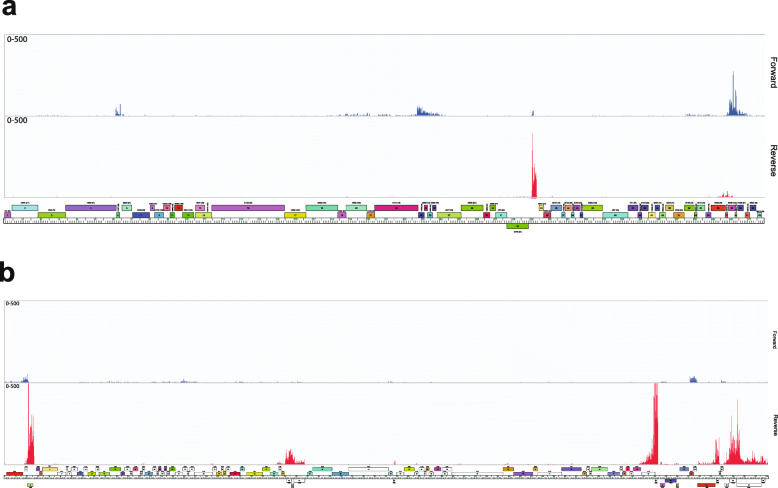
Lysogenic gene expression profiles of prophages **a.** BPs and **b.** McProf from the *M. chelonae* WT strain. RNAseq profiles are shown for forward (blue) and reverse (red) DNA strands. Note that in **a.** the sequence reads are mapped to the viral BPs genome rather than prophage genome whereas in **b.** the sequence reads are mapped to the McProf prophage genome. The number of reads mapped are on the y-axis and the genome maps are shown below. Genes expressed from the McProf prophage genome in the double lysogen were not significantly different from the WT strain and therefore the expression profile was not included

To determine if the presence of BPs alters gene expression from the McProf prophage genome, differential expression of McProf genes was examined between the WT strain (McProf) and the double lysogen (BPs, McProf). None of the expressed McProf genes were significantly differentially expressed in the presence of the BPs prophage (FC > 1.99 and FDR < 0.05). Because there was no difference in expression profiles, we present below the expression profile of only the McProf prophage from the *M. chelonae* WT (McProf) strain (Fig. 8b).

The immunity repressors from both the BPs (gp33) and McProf (gp3) genomes are highly expressed during lysogeny of *M. chelonae* (Fig. 8a)*.* The BPs genome also expresses, gp58, a gene of unknown function that is part of a mycobacteriophage mobile element (MPME1) (Fig. 8a) [28]. There are an additional 15 genes expressed at varying levels from the McProf genome (Fig. 8b). The integrase (gp1) is expressed at low levels and is adjacent to a moderately expressed genes of no known function (gp2). There are three reverse oriented genes, gp48–50, located between the HNH endonuclease (gp47) and the small subunit terminase (gp51) and a small reverse oriented gene (gp56) adjacent to the scaffolding protein with moderately and low expression, respectively. We were not able to determine functions for these genes; however, gp48 and gp49 do have predicted membrane domains. The remaining genes expressed from the McProf prophage genome are located between the structural genes and *attR* and many do not have predicted gene functions, including the most highly expressed McProf gene, gp84. There is also strong expression from the gene cassette containing the putative WXG-100 family polymorphic toxin and immunity protein (gp96–98). None of the expressed McProf genes were significantly differentially expressed in the presence of the BPs prophage (FC > 1.99 and FDR < 0.05).

## Discussion

The incidence of non-tuberculosis mycobacterial disease has increased over the last 20 years [40]. *M. abscessus*, along with *M. avium*, is the major cause of broncho-pulmonary infections in cystic fibrosis patients, and is of increasing concern due its high levels of intrinsic antibiotic resistance [9]. CLA and AMK are the two core drugs used to treat *M. abscessus* infections; however, development of resistance to these drugs is common during treatment [9]. Mycobacterial resistance to CLA and AMK are often the result of mutations in 23 s rRNA or 16 s rRNA, respectively, but mutations alone do not completely account for AMK and CLA resistant phenotypes in *M. abscessus* clinical isolates [7, 13]. Induction of the *whi*B7 regulon in mycobacteria is the second major contributor to resistance to AMK and CLA [7]. Heightened expression of *whi*B7 is consistently observed in extensively resistant *M. abscessus* isolates relative to drug susceptible isolates [7, 13]. Understanding the mechanisms that drive increased expression of *whi*B7 will be important for improving treatment of resistant mycobacterial infections. The WhiB7 response is highly conserved across all mycobacteria and our studies shows that the WhiB7 regulon in *M. chelonae* overlaps considerably with that of *M. abscessus* [12, 15]. Here we describe for the first time that prophages in mycobacteria alter antibiotic resistance and expression of intrinsic antibiotic resistance genes of the *whi*B7 regulon. This study provides valuable insight into the role prophages play in mycobacterial antibiotic resistance and novel mechanisms of *whi*B7 induction.

In this report we show for the first time that prophages in mycobacteria can contribute to increased resistance to aminoglycosides AMK and TOB (Table 1; Fig. 1; and Fig. 2). This is similar to the resistance observed in *E. coli* carrying multiple prophages [1]. Of the two prophages investigated in this study, the naturally occurring prophage, McProf, appears to play the more important role in inducing intrinsic resistance in *M. chelonae* (Table 1; Fig. 1; and Fig. 2). Strains carrying the McProf prophage demonstrated increased AMK resistance relative to the non-lysogen and BPs single lysogen strains in the absence of ACI. The increased AMK resistance observed in McProf carrying strains was further increased by either ACI treatment or the presence of a second prophage, BPs. Enhanced resistance in McProf-carrying strains in response to ACI treatment was also observed in the TOB MIC assays. We know that sub-inhibitory concentrations of ribosome targeting antibiotics, such as ACI, induce *whi*B7 expression and intrinsic drug resistance and we observed this in the non-lysogen (ΔMcProf) strain (Fig. 2) [14, 15]. The presence of McProf appears to enhance the effect of ACI on resistance in the AMK and TOB assays (Figs. 2 and 3).

The dramatically higher *whi*B7 expression in the strain carrying both prophages (BPs, McProf) likely contributes to the heightened AMK and TOB resistance observed in the double lysogen (Figs. 2 and 4; Tables 4 and 5). We observed high *whi*B7 expression in the double lysogen in the absence of antibiotics or other conditions known to induce *whi*B7, revealing prophage as a novel mechanism of inducing *whi*B7 expression and intrinsic antibiotic resistance. Also upregulated in the double lysogen are *whi*B7 regulon genes that can explain the increased resistance to aminoglycosides AMK and TOB [41]. The GNAT acetyltransferase, *eis2,* and *tap,* a multidrug efflux pump, were each upregulated ~ 10-fold in the double lysogen and confer resistance to aminoglycosides [12, 42, 43]. 2′-N-acetyltransferase AAC (2′) also contributes to resistance to TOB and AMK, however this gene (BB28_RS22055) was not significantly upregulated in our data set [44]. It is possible that other N-acetyltransferases (BB28_RS23100, BB28_RS01940, BB28_RS14560), aminoglycoside phosphotransferases (BB28_RS12685), or potential efflux pumps (BB28_11540) upregulated in our dataset contributed to AMK and TOB resistance of the double lysogen (Table 5). Tap, along with TetV (BB28_RS13560) target tetracycline efflux, however, we did not consistently see an increase in TET resistance across all our MIC trials [45, 46]*.* Although *M. chelonae* lacks an *erm* gene, there was a slight elevation in the *whi*B7 regulon gene, *hflx,* but this did not result in a significant change in CLA resistance [32].

Other mechanisms of intrinsic resistance also likely contribute to the AMK and TOB resistance observed in the McProf-carrying strains. The McProf-carrying strains demonstrated the highest AMK resistance in the presence and absence of ACI treatment. Although *whi*B7 expression was highest in the double lysogen (BPs, McProf) treated with ACI among the four strains, *whi*B7 expression levels in the single McProf strain did not correlate with its relative AMK resistance among the four strains. AMK is also a potent inducer of *whi*B7 expression in mycobacteria; however, AMK had little to no effect on *whi*B7 expression in strains carrying McProf [12]. The strong induction of *whi*B7 with AMK treatment in strains that lack McProf suggests that the presence of the McProf prophage may affect cell wall permeability. A decrease in cell wall permeability in McProf-carrying strains would likely contribute to the observed AMK resistance in these strains. Investigating differences in cell wall permeability in the presence and absence of McProf will be important for understanding the effect of prophages on drug resistance.

We do not yet know how the two prophages, BPs and McProf, interact to alter *whi*B7 expression in the double lysogen strain. The McProf genome appears to only express genes through lysogenic infection of *M. chelonae*, whereas BPs can carry out lysogenic and lytic infection (via induction) in a population of double lysogen cells. It’s therefore possible that either lytic or lysogenic gene expression from BPs interacts with any of the 16 genes products expressed from the McProf genome through an unknown mechanism to alter *whi*B7 expression. Each of the 16 expressed McProf genes will be investigated for a potential role in altered *whi*B7 expression; however, the genes in the WXG-100 family polymorphic toxin cassette are strong candidates. Activated toxin systems could potentially act as a trigger to the WhiB7 stress response. Sub-inhibitory concentrations of antibiotics and BPs phage infection both enhanced antibiotic resistance in *M. chelonae* in the presence of prophage McProf (Figs. 1 and 4), conditions also known to activate toxin/antitoxin systems [47–51]. Toxin/antitoxin systems are also known to function as stress response modules and regulators of adaptive responses to stresses associated with host environment and drug treatment [49]. Further, toxin/antitoxin systems are abundant in pathogenic mycobacteria and are more highly expressed in the most virulent strains of *M. tuberculosis* [49]. In comparison there are relatively few toxin/antitoxin systems in non-pathogenic mycobacteria [47]. Toxin/antitoxin systems also stabilize replicative elements (e.g. plasmids and prophage) and defend against phage lytic infection [52, 53]. The increased stability of the BPs prophage in the presence of McProf compared to BPs lysogens in the cured *M. chelonae* (ΔMcProf) and its reported instability in *M. smegmatis* strains [28, 29] suggests that such system encoded by McProf is active.

## Conclusions

We have established that dramatic increases in *whi*B7 expression and AMK resistance only occurs in *M. chelonae* strains carrying a type of prophage that is also found naturally in *M. abscessus* strains. The observed AMK resistance in the presence of prophage McProf is further enhanced by exposure to sub-inhibitory concentrations or by the presence of a second prophage, BPs. Pathogenic mycobacteria typically carry one or more prophages that are capable of induction and in infected tissues are likely exposed to lytic phage infection and sub-inhibitory concentrations of antibiotics during treatment. Our novel research findings indicate that prophage could be drivers of important intrinsic antibiotic resistance genes in response to such stresses. To determine the mechanism by which phage alter intrinsic antibiotic resistance in mycobacteria, we are exploring the function and impact of specific phage genes on expression of *whi*B7 in the presence of various environmental stressors.

## Methods

### Bacterial and viral strains

*Mycobacterium chelonae* (ATCC®35,752, American Type Culture Collection, Manassas, VA) was cultivated at 30 °C with shaking at 200 RPM in liquid Middlebrook 7H9 (BD, Sparks, MD) supplemented with 10% ADO (Bovine Serum Albumin, Dextrose, Oleic Acid) in the absence of antibiotics unless indicated. Tween80 to a final concentration of 0.05% was added to the media to avoid clumping but was omitted in MIC experiments and any experiments involving phage infection. The wildtype (WT) strain of *M. chelonae* carries a prophage that we have named McProf and we refer to this strain as *M. chelonae* (McProf) (Table 2). Cloning was carried out in chemically competent *Escherichia coli* DH5α (New England Biolabs (NEB), Ipswich, MA). Kanamycin was used for selection of the expression vector pST-KT at 250 μg ml^− 1^. Strains used in this study are listed in Table 2.

Bacteriophage BPs was obtained from the Hatfull Laboratory [28]. Phage lysates were propagated through plaque assays in either *M. smegmatis* MC^2^155 or *M. chelonae* (McProf), or a cured strain of *M. chelonae* that we refer to as the non-lysogen *M. chelonae* strain (ΔMcProf) (Table 2) [54]. Briefly, 0.5-ml aliquots of late-log phase bacteria were incubated with serially-diluted phage samples for 15 min before plating in 4.5 ml of 7H9 top agar containing 0.45% agar onto 7H10 agar plates. Phage stocks were created by flooding plates with nearly confluent bacterial lysis with phage buffer (10 mM Tris/HCl pH 7.5, 10 mM MgSO_4_, 1 mM CaCl_2_, 68.5 mM NaCl).

### Curing of M. chelonae WT strain

A recombinant strain of *M. chelonae* (McProf) that overexpresses the McProf excision (*xis*) gene (McProf gp5) was created by cloning a 292-bp G-block (Integrated DNA Techonologies, Coralville, IA) encoding the *xis* gene into the mycobacterial expression vector, pST-KT using Gibson Assembly (NEB, Ipswich, MA) [30]. Recombinant plasmids were sequenced to verify the presence of the *xis* sequence prior to electroporating into competent WT *M. chelonae* (McProf) [55]*.* Cultures of recombinant *M. chelonae* carrying pST-KT_*xis* were grown in 10-mL volumes for 48 h at 30 °C with shaking. Optical density was measured at a wavelength of 600 nm, and samples sub-cultured to an optical density of 0.05. Cultures were then grown to an optical density of 0.6 and treated with 500 μg mL^− 1^ of anhydrotetracycline (ATc) prepared in dimethylsulfoxide (DMSO) or an equivalent volume of DMSO. Cultures were incubated at 30 °C with shaking for an additional 72 h. A 0.5-mL sample of each culture was harvested and serially diluted in 7H9-OAD. Dilutions were plated onto 7H10-OAD supplemented with 250 μg mL^− 1^ of kanamycin in 100-μL volumes and incubated at 30 °C for 5 d. Resulting colonies were PCR screened for the loss of prophage McProf using a set of four primers that amplify either the bacterial *attB* site, indicating loss of the prophage, or the attachment junctions *attL* and *attR*, indicating the presence of the prophage (Table 3). The *attB* PCR product was sequenced to confirm the clean excision of the prophage.

### Isolation of lysogenic strains

Lysogens were isolated by plating serially diluted *M. chelonae* strains in 4.5 ml of 7H9 top agar onto 7H10 agar treated or not treated with 10^9^ PFUs of BPs. After 6 d of incubation at 30 °C, colonies were picked and screened for properties indicative of lysogens, including release of phage particles into culture supernatant, superinfection immunity to BPs infection and PCR detection of prophage attachment sites, *att*L and *att*R (Table 3). The efficiency of lysogeny was determined by dividing the number of colonies present on virus-treated plates by the number of colonies present on un-treated plates and multiplying by 100. Genomic DNA from BPs lysogens of the WT *M. chelonae* strain (referred to as *M. chelonae* double lysogen (BPs, McProf)) (Table 2) were sequenced to confirm the presence of the BPs genome in the *M. chelonae* genome. Whole genome libraries were generated by Genome Technologies at Jackson Laboratory (Bar Harbor, ME) and sequenced on one 2X150-bp MiSeq sequence run. Sequence reads were assembled by aligning reads to a reference genome and reads that did not map to the host genome were assembled de novo.

### RNA isolations

Total RNA was isolated from six replicates of 4-ml samples of *M. chelonae* grown to an OD_600_ of 1.0. Cultures were treated with RNAProtect Bacteria Reagent (Qiagen, Germantown, MD) before centrifuging at 5000 x *g* for 10 min. Cell pellets were resuspended in 100 μl of TE containing 100 μg ml^− 1^ lysozyme and incubated at room temperature for 40 min. After adding 700 μl of RLT buffer (Qiagen), cells were transferred to 2-ml Lysing Matrix B tubes (MP Biomedicals, Irvine, CA) and homogenized in ice-cold adaptors in the TissueLyser LT (Qiagen) set for 8 min at 50 Hz. RNA extractions were carried out on the lysates using the RNeasy Mini Kit (Qiagen) with DNAse treatment (Qiagen) on the column according to the manufacturer’s recommendations. After elution of RNA in 50 μl of water, samples were treated with a second application of DNAse using the Turbo DNA-free Kit (Thermo Scientific, Waltham, MA) according to the manufacturer’s recommendations. The quantity of RNA was determined with the NanoDrop ND-1000 spectrophotometer (NanoDrop Technologies, Montchanin, DE, USA). The quality of RNA was determined by gel electrophoresis using the FlashGel RNA system (Lonza, Rockland, ME).

In the ACI and AMK induction experiments, RNA was isolated as described above with the following modifications. Cultures were grown to an OD_600_ of 0.7 or 0.9 and treated with 75 μM ACI for 3 h or with 16.7 μM AMK for 1 h, respectively, before harvesting cells for RNA. Untreated control cultures were incubated for equivalent amount of time prior to harvesting cells.

### RNAseq

RNA used in RNAseq experiments was sent to the Delaware DNA Sequencing and Genotyping Center (Newark, DE) for quality control analysis, library preparation and paired end sequencing on the Illumina HiSeq 2500. Read length was set to 51 bases with the samples run on two separate lanes. Raw sequencing data files were uploaded to the public Galaxy server *at* usegalaxy.org [56]. Read files from the two lanes were concatenated and read quality was determined using FastQC [57]. The reads were processed using the Trim Galore! with the FastQC output as a guide [58]*. Retained reads had a quality score minimum of 30, and with the first 9 bases on the 5′ end and the last base on the 3′ end removed. Though rRNA was depleted prior to sequencing, we discovered that depletion of rRNA was incomplete. The rRNA reads were computationally removed by alignment to the M. chelonae rRNA operon using BowTie2 and saving the non-aligned reads* [59]*.* Average number of reads per sample was 2,280,954 reads for the *M. chelonae* (McProf) samples and 2,484,260 reads for the double lysogen (BPs, McProf) samples This gave us an average read depth of 40 reads per base for the transcriptome of both the WT (McProf) and double (BPs, McProf) lysogen samples. *Processed reads were then quantitated using Salmon* [60] *by aligning in a strand-specific orientation to the M. chelonae* (CCUG 47445) transcriptome *using a coding transcript fasta (GenBank). The alignment was adjusted for the high GC content of the mycobacterial genome. Mate pair 1 was specified as coming from the reverse strand (SR). Strand specificity was necessary because reads from two convergent genes often overlapped.*

*Output from Salmon quantification was used for pairwise comparisons of expression, using the R statistical package DESeq2* [61] *and the NumReads values as described by the authors. Genes with low expression levels (reads < 10) were removed. Genes were considered significantly regulated if Log2 fold change (Log2FC) was greater than 1.0 and the False Discovery Rate (FDR) was less than 0.05.* Although the *M. chelonae* genome is sequenced and has an annotation, the gene functions are poorly characterized. We therefore generated a table of *M. chelonae* genes with orthologs in *M. tuberculosis, M. abscessus* and *M. smegmatis* using the OrthoDB pipeline, a series of scripts from OrthoDB [62]. This gave us the best alignment between the three genomes and together with blastP on MycoBrowser and HHpred, helped identify numerous significantly upregulated *M. chelonae* genes with potential virulence functions [62–64]. The RNAseq data set was validated in two independent RNA isolation experiments using qPCR assays that quantified expression of upregulated (*whi*B7 and *tap*) and downregulated genes (glycerol kinase (*glp*K)) from the RNAseq data set (data not shown).

### RTqPCR

cDNA was synthesized from 500 ng of total RNA in 20-μl reactions containing qScript cDNA Supermix (Quantabio, Beverly, MA) according to the manufacturer’s recommendations. Reactions were incubated for 5 min at 25 °C, 20 min at 42 °C and heat inactivated at 85 °C for 5 min. cDNA was diluted 1:6 in 10 mM Tris and stored at − 20 °C.

Real-time PCR assays were performed using the Bio-Rad CFX96 Real-Time system (Bio-Rad Laboratories, Hercules, CA). Using Primer3 software, primer sets were designed to amplify a 100-bp sequence in the gene of interest (Table 6). Quantitative PCR (qPCR) was carried out in triplicate 25-μL reactions containing 200 nM gene-specific primers (Table 6), 1 μl diluted cDNA (1:5) and PerfeCTa SYBR Green Supermix (Quantabio), according to manufacturer’s instructions. Reactions were incubated at 95 °C for 3 min, followed by 40 cycles of 95 °C for 10 s and 60 °C for 30 s. A melt curve analysis was performed to confirm that only one amplicon was created by each primer set. The change in abundance of gene-specific RNA was normalized to *M. chelonae 16 s rRNA* and calculated using the 2^-∆∆CT^ method [65]. Positive and no-template controls were included in real-time PCR analysis.

**Table 6:** qPCR primers used in this study

Target	Primers	Sequence (5' to 3')	Tm (˚C)	% GC	amplicon size (bp)
Forward and reverse primers targeting *whi*B7 (BB28_RS17590)	WhiB7_qPCR_F4	ACTTTCCGCGAACCACAG	55.6	55.6	81
	WhiB7_qPCR_R1a	ATGATGACCGTCGAAGTGG	54.6	52.6	
Forward and reverse primers targeting *16s rRNA* (BB28_RS07070)	Myco_16S_F1	CCGGATAGGACCACACACTT	56.6	55	91
	Myco_16S_R1	ATTACCCCACCAACAAGCTG	55.4	50	

### Minimum inhibitory concentration determination

Minimum inhibitory concentration (MIC) assays were performed according to Burian et al. (2012) and Ramon-Garcia et al. (2013) [14, 66]. Briefly, cultures were grown for 2 d in 7H9 supplemented with OAD and then sub-cultured such that overnight incubation at 30 °C with shaking allowed cultures to reach an OD_600_ of 0.1–0.3. Cultures were diluted to a density of 10^5^ cells ml^− 1^ and applied in 50-μl volumes to wells of a 96-well plate containing 50 μl of 7H9 media with antibiotic concentrations that varied by 2-fold dilutions across the plate. Because clarithromycin was prepared in DMSO, an equivalent amount of DMSO was included in all wells. Each strain was tested at each antibiotic concentration in replicates of six and no-antibiotic controls were performed in replicates of 16. Inoculated plates were sealed with porous adhesive culture plate films (VWR International, Radnor, PA), wrapped with parafilm and incubated at 30 °C for two d before adding 1 μl (assays presented in Figs. 1 and 2) or 2 μl (assays presented in Fig. 3) of AlamarBlue (BioRad, Hercules, CA) and 25 μl of 25% Tween80 to each well. After incubation at 30 °C for 1 d, the MIC was determined as the lowest drug concentration that completely inhibited growth. Viability was also determined by measuring the optical density at 570- and 600 nm and the percent viability of cells was calculated as the percent difference in reduction between antibiotic-treated cells and untreated cells according to the manufacturer’s instructions. Each assay was replicated in at least three independent experiments.

### McProf genome analysis

The McProf genome was detected in the *M. chelonae* genome using Phaster [67]. The genome ends were defined as *attL* and *attR* and the sequence was annotated using DNA Master (http://cobamide2.bio.pitt.edu) and PECAAN (https://pecaan.kbrinsgd.org/index.html). Genes were identified and gene start coordinates determined first by auto-annotation using Glimmer and GeneMark, then by manual inspection of each predicted gene [68, 69]. Gene functions were predicted using HHPRED and BLAST [70, 71]. Genome map representations were created in Phamerator using database McProf_DB [34].

## Data Availability

The RNAseq data set analyzed during this study is deposited in the Gene expression Omnibus (GEO) with the accession number GSE164210, https://www.ncbi.nlm.nih.gov/geo/query/acc.cgi?&acc=GSE164210.
